# Effect of the long-term wearing of spectacle lenses with highly aspherical lenslets on visual field sensitivity in children with myopia

**DOI:** 10.1186/s12886-026-04781-1

**Published:** 2026-03-31

**Authors:** Zaifeng Cui, Huayu Zhang, Xiao Fang, Yingying Huang, Jinhua Bao, Xue Li

**Affiliations:** National Clinical Research Center for Ophthalmology and Optometry, Eye Hospital, Wenzhou Medical University, 270 West Xueyuan Road, Wenzhou, Zhejiang 325027 China

**Keywords:** Highly aspherical lenslets (HAL), Myopia control, Perimetry, Visual field sensitivity, Children

## Abstract

**Introduction:**

This study investigated the effects of the short and long-term wearing of spectacle lenses with highly aspherical lenslets (HAL) on visual field sensitivity in children with myopia.

**Methods:**

This prospective case‒control study included 80 children with myopia aged 13–18 years, whose spherical equivalent refractive error (SER) ranged from ﹣1.00 D to -7.25 D. Forty children wore single vision lenses (SVL) habitually, while the remaining 40 children had been wearing HAL lenses for five years. Static visual field sensitivity was assessed using automatic static perimetry (Goldman III target) involving 76 white light points within a 30° radius visual field. After a practice round without the test lenses, measurements were taken while the participants wore either test HAL lenses or SVL in a randomized order. All assessments were conducted on the right eye while the left eye was occluded.

**Results:**

Statistical analysis using generalized estimating equations (GEEs) revealed no significant main effects of group (*p* = 0.17) or lens (*p* = 0.52), nor significant interaction effect between these factors (*p* = 0.81). When the test SVL were used, the difference in mean sensitivity between the HAL and SVL groups (HAL group minus SVL group) was 0.7 ± 1.19 dB (ranging from ﹣ 2.5 dB to 4.8 dB). When the test HAL lenses were used, a mean difference of 0.7 ± 0.91 dB (ranging from ﹣ 0.6 dB to 3.3 dB) was observed between the two groups. Visual field reliability parameters (fixation, false-positive, and false-negative error ratios) were similar between conditions (all *p* > 0.05).

**Conclusion:**

Neither short nor long-term wearing of spectacles with HAL affected full-field static visual field sensitivity within the 30° radius visual field in children with myopia aged 13 to 18 years, supporting their safety profile in controlling myopia.

**Supplementary Information:**

The online version contains supplementary material available at 10.1186/s12886-026-04781-1.

## Introduction

The prevalence of myopia has been increasing in recent decades, making this condition a global public health concern [1, 2]. As myopia progresses, the risk of high myopia-related complications such as myopic macular degeneration, retinal detachment, cataracts, and open angle glaucoma increases substantially, resulting in irreversible visual impairment [3]. 

Currently, various optical approaches based on peripheral defocus, including orthokeratology (OK) lenses, multifocal soft contact lenses, and myopia management spectacle lenses, are widely used in clinical practice to slow myopia progression [4–11] These inventions for myopia management alter the blurred signals affecting the peripheral retina, which may impact visual performance among children. Short-term wearing of spectacle lenses with highly aspherical lenslets (HAL) causes a slight impairment to central vision compared to single vision lenses (SVL), while prolonged wearing improves high-contrast visual acuity and stereoscopic vision, resulting in no significant difference between the HAL and SVL groups, thereby mitigating the potential negative impact on children’s visual quality [12]. 

The impact of HAL lenses on peripheral visual functions has been explored in some studies. Gao et al. found that peripheral vision function, including peripheral contrast sensitivity, peripheral motion perception and useful field of view, were not affected by HAL lenses in adults [13]. In contrast, Wu et al. reported that HAL lenses increased the peripheral coherence threshold in children, suggesting interference with peripheral visual processing [14]. Both studies examined the immediate effects of HAL lenses wearing, while different conclusions were drawn based on distinct study populations, which indicated that children may exhibit different visual responses related to the ongoing maturation of their peripheral visual system.

Gao et al. reported only minimal, clinically insignificant changes in static visual field sensitivity in adults wearing HAL lenses compared with SVL [15]. Considering that myopia control is a long-term process and that neural plasticity allows the visual system to adapt to sustained optical conditions, it remains unclear whether prolonged wear of HAL lenses affects visual field sensitivity in children. Therefore, this study compares the static visual field sensitivity of children who have been long-term wearers of HAL lenses and SVL for myopia correction using automated static perimetry.

## Methods

### Participants

Long-term HAL lenses wearers were recruited from participants who completed a 5-year clinical trial evaluating the effects of continuous HAL lenses wear on myopia progression (ChiCTR2100047262) [4]. Potentially eligible children with myopia in the SVL (control) group were recruited from the Eye Hospital, Wenzhou Medical University, and were matched to those in the HAL group in terms of age, spherical refraction equivalent (SER), and best-corrected visual acuity (BCVA). Those in the SVL group wore SVL habitually and had no history of myopia control interventions during the past year. The visual acuity of the right eye had to be equal to or better than 0.05 logMAR with their habitual corrective lenses. This prospective case–control study adhered to the tenets of the Declaration of Helsinki and was approved by the Ethics Committee of the Eye Hospital, Wenzhou Medical University (No. 2023-079-K-66-01). Written informed consent was obtained from the children and their legal guardians prior to the study.

### Test lenses

Two test lenses were evaluated: (1) the HAL lens, which has 11 circular, highly aspherical lenslets arranged in concentric circles, and a clear central 9 mm diameter region to provide clear distance vision; [16] and (2) the traditional single vision spectacle lens (SVL). Each test lens was mounted in a 36.5 mm diameter trial lens ring to position it in the lens holder of the perimeter, with the optical center of the test lens coincident with the geometric center of the trial lens ring.

### Visual field measurement

An automatic static visual field test was performed using an Optical Kinetic Perimeter (SK-950B; Chongqing Sunkingdom Medical Instrument Co., Ltd., China) with a 30 − 2 threshold program. The test was a modified procedure based on the study by Gao et al., as previously described in detail [15]. The test measured the brightness sensitivity to white static stimuli at 76 points in the 30° radius visual field. The visual stimulus was a Goldman III target (0.43°), which appeared for 0.2 s at different positions in the visual field in a random order.

Before the test, the participants’ birth dates and refraction data were entered into the perimeter to automatically calculate the lens prescription needed to compensate for the 30 cm test distance to minimize potential reductions in sensitivity attributable to accommodative insufficiency–induced defocus [17]. The corrective lenses and test lenses were placed in a lens holder, which was positioned as close to the eye as possible without touching the eyelashes to minimize frame edge interference. The tests began immediately after the test lenses were worn, without any adaptation period.

The participants’ heads were stabilized using chin and forehead rests during testing. The participants were instructed to fixate on the central orange light and click on the button whenever they detected a flash. A blind spot check was performed to verify proper fixation throughout testing. During the test, the device continuously monitored the participant’s eye position to ensure the reliability of the results. A practice trial was given to familiarize participants with the procedure. The perimeter monitored the pupil size and eye position continuously, and automatic adjustments were made as needed. HAL lenses and SVL were tested in random order. Each session lasted 4–5 min, with a 2-minute break between sessions. The right eye was tested with the left eye occluded to ensure standardization and consistency of the experiment.

### Data collection and statistical analysis

A test result was deemed ineligible if it exhibited any of the following: a false-positive rate exceeding 15%, a false-negative error greater than 20%, or more than 20% fixation loss. All analyses were performed with SPSS 27.0 software. The Kolmogorov‒Smirnov test was used to test the normality of the data. For the analysis of baseline demographic and ocular characteristics, variables that followed a normal distribution with homogeneity of variance were analyzed using an independent samples t-test, whereas variables that did not meet the normality assumption were analyzed using the Mann–Whitney U test. Since the threshold data failed to meet normality and some ineligible data needed to be excluded, generalized estimating equations (GEEs) were employed to analyze repeated sensitivity measurements across the 76 visual field locations under different group and lens conditions, with Bonferroni correction applied to account for multiple comparisons. Statistical significance was indicated if *p* < 0.05.

## Results

The study included 80 participants, with 40 in the HAL group and 40 in the SVL group. There were no significant differences in age, sex, SER, or BCVA between groups (all *p* > 0.05; Table 1). Table 2 lists detailed information on the participants included in the analysis (i.e., after exclusion of those with ineligible test results). The reliability parameters and test duration did not differ significantly between the HAL and SVL groups or between the use of the test lenses, HAL lenses and SVL (Table 3).

**Fig. 1:**
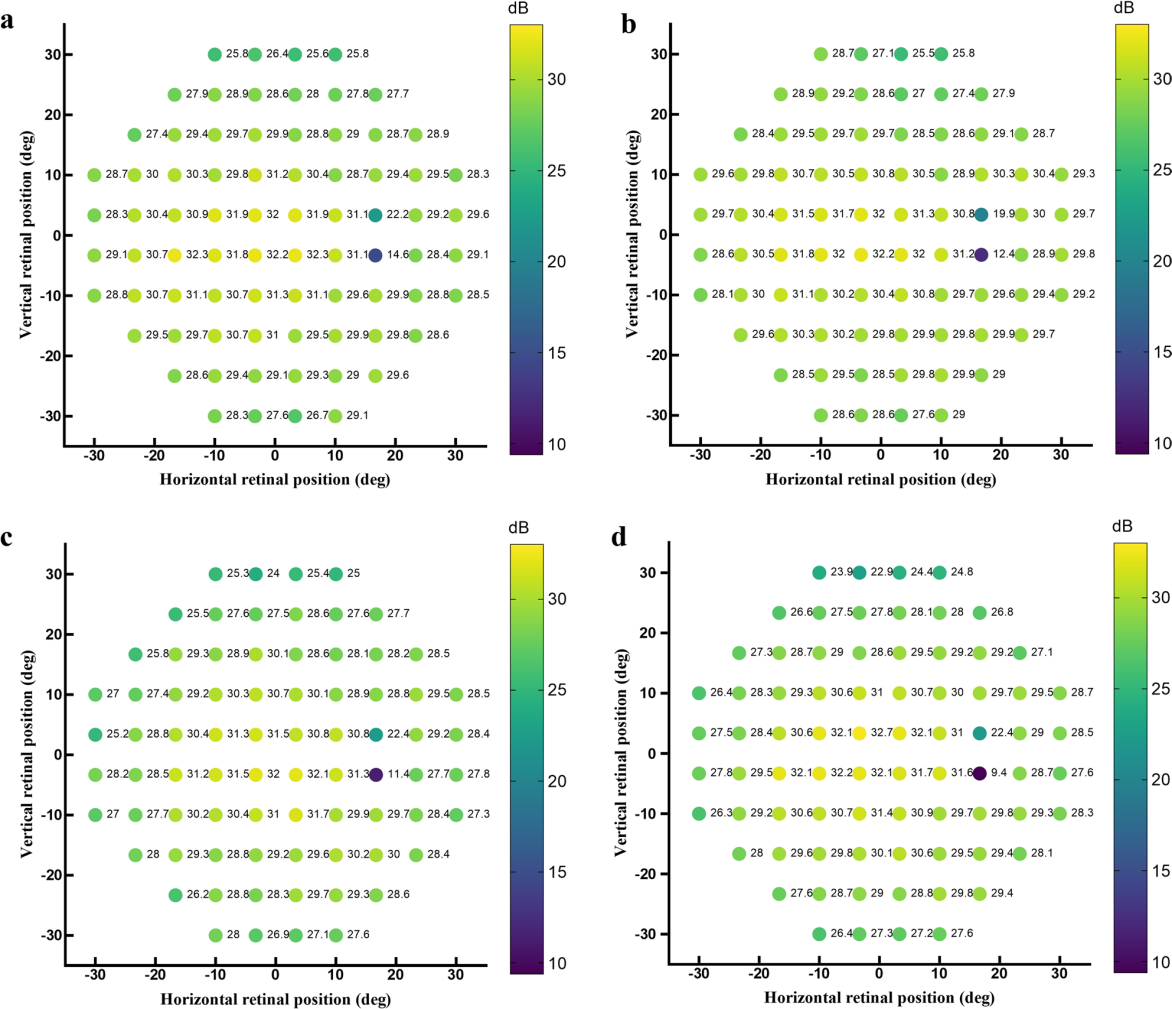
Sensitivity maps of HAL and SVL groups. Panels (**a**) and (**b**) show the raw sensitivity data of the HAL group wearing HAL lenses (**a**) and SVL (**c**), respectively. Panels (**c**) and (**d**) show the raw sensitivity data of the SVL group wearing HAL lenses (**c**) and SVL (**d**), respectively. The color scale shows the mean sensitivity values of each point. HAL: spectacle lenses with highly aspherical lenslets; SVL: single vision lenses

**Fig. 2:**
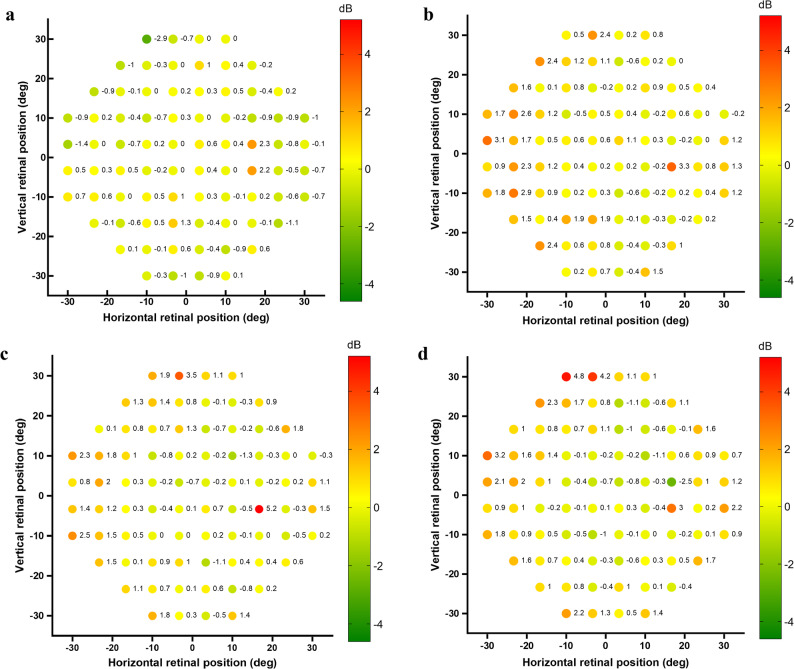
Sensitivity difference maps. Panel (**a**) shows the mean differences for HAL wearers with HAL lenses vs. SVL. Panel (**b**) shows the mean differences for HAL wearers with HAL lenses vs. SVL wearers with HAL lenses. Panel (**c**) shows the mean differences for HAL wearers with HAL lenses vs. SVL wearers with SVL. Panel (**d**) shows the mean differences for HAL wearers with SVL vs. SVL wearers with SVL. Positive values indicate higher sensitivity in the former condition than in the latter, whereas negative values indicate lower sensitivity. HAL: spectacle lenses with highly aspherical lenslets; SVL: single vision lenses

The results revealed that the main effects of group (Wald χ² = 1.864, *p* = 0.17) and lens (Wald χ² = 0.405, *p* = 0.52), as well as the group × lens interaction (Wald χ² = 0.057, *p* = 0.81), were not statistically significant. The average raw sensitivity data of the HAL and SVL groups wearing the test HAL lenses and SVL are shown in Fig. 1, and the average differences are shown in Fig. 2. The sensitivity threshold differences for HAL wearers with HAL lenses vs. SVL ranged from ﹣2.9 dB to 2.3 dB across the 76 test points. Similarly, for HAL wearers with HAL lenses vs. SVL wearers with HAL lenses, the threshold differences ranged from﹣0.6 dB to 3.3 dB. For HAL wearers with HAL lenses vs. SVL wearers with SVL, the threshold differences ranged from ﹣1.3 dB to 5.2 dB. Lastly, for HAL wearers with SVL vs. SVL wearers with SVL, the threshold differences ranged from ﹣2.5 dB to 4.8 dB. A positive value indicates that the sensitivity of the former condition in the comparison was higher than that of the latter, whereas a negative value indicates that the former condition had lower sensitivity than the latter. To explore whether differences might be more prominent in the peripheral visual field, an additional analysis was conducted for 44 test locations between 20° and 30° eccentricity, corresponding to the visual field covered by the lenslets. Within this peripheral subset, GEE analysis likewise revealed no significant main effects of group or lens type, nor any significant interaction effects (all *p* > 0.05).

A comparison of the visual field test reliability parameters and test duration under different lens conditions is presented in Table 3. The results revealed that the fixation error ratio, false positive error ratio, and false negative error ratio did not differ significantly with respect to the group effect, lens effect, or group × lens interaction (all *p* > 0.05). The HAL group tended to complete the test faster than the SVL group did, with the group effect statistical significance (*p* = 0.048). Further details on the visual field–related parameters are available in Additional file 1.

**Table 1:** Baseline demographic and ocular characteristics of the participants in each group

	HAL group (*n* = 40)	SVL group (*n* = 40)	*P* value
Age (y)^a^	15.70 ± 1.14 (13~17)	15.6 ± 1.53 (13~18)	0.60
Sex			0.26
Male, n (%)	19(47.5%)	14(35%)	
Female, n (%)	21(52.5%)	26(65%)	
SER (D)^b^	-4.13 ± 1.82	-3.93 ± 1.43	0.53
BCVA (logMAR)^a^	-0.090 ± 0.038	-0.09 ± 0.062	0.90

Data are presented as the means ± SDs, unless stated otherwise

HAL: spectacle lenses with highly aspherical lenslets; SVL: single vision lenses; SER: spherical equivalent refraction; D: diopters; BCVA: best-corrected visual acuity

^a^ The Mann–Whitney U test was employed

^b^ The independent samples t-test was employed

**Table 2:** The number of valid samples for the HAL group and SVL group wearing two lenses

Test lens\Group	HAL	SVL
HAL group (n)	27	25
SVL group (n)	33	32

HAL: spectacle lenses with highly aspherical lenslets; SVL: single vision lenses

**Table 3:** Comparing visual field parameters and test time between HAL and SVL groups

		HAL test lens	SVL test lens	*p* values		
				Groups	Test lens	Group*Test lens
FixationError Ratio (%)	HAL group	8.48%±13.44%	6.75%±13.74%	0.70	0.96	0.10
	SVL group	6.23%±12.19%	7.86%±10.69%			
FalsePosError Ratio (%)	HAL group	3.33%±0.84%	2.26%±0.81%	0.54	0.73	0.27
	SVL group	2.05%±0.63%	2.61%±0.74%			
FalseNegError Ratio (%)	HAL group	5.40%±1.82%	5.69%±1.45%	0.32	0.51	0.69
	SVL group	7.02%±1.75%	8.24%±1.70%			
TestDuringTime (min)	HAL group	4.54 ± 0.51	4.52 ± 0.54	0.048	0.85	0.69
	SVL group	4.83 ± 0.75	4.83 ± 0.71			

Data are presented as the median ± interquartile range

HAL: spectacle lenses with highly aspherical lenslets SVL: single vision lenses

## Discussion

In this study, the impact of the initial and long-term wearing of HAL lenses on static visual field sensitivity in children with myopia was assessed. Using HAL lenses for myopia management did not affect static visual field sensitivity, either in the long term or in the initial stage. Therefore, this study provides further evidence supporting the safety of the long-term wearing of HAL lenses.

The impact of myopia management lenses on visual field sensitivity has not been studied extensively, but full-field or continuous peripheral defocus impairs visual field sensitivity in adults. For example, + 1.00 D ~ + 4.00 D full defocus reduced contrast sensitivity for stimuli equal to or smaller than the Goldman target III within a visual field of 30–40 degrees in adults [18], and + 2.00 D full defocus decreased sensitivity in kinetic perimetry in myopic adults [19]. A study investigating center-distance multifocal contact lenses used for presbyopia correction demonstrated a general decrease in measurements with the Humphreys 24 - 2 Swedish interactive threshold algorithm (SITA) standard automated perimetry (SAP) [20]. 

Research on the impact of lenses with discontinuous defocus designs (e.g., myopia management lenses with lenslets) remains scarce. Compared with SVL, HAL lenses impair children’s central vision acuity slightly while allowing adaptation, and they do not alter accommodation or phoria significantly in children with myopia [12]. Moreover, the short-term wearing of HAL lenses in adults does not reduce visual sensitivity across the visual field [15]. No statistically significant difference in sensitivity was demonstrated in the comparison between DIMS and SVL following one week of adaptation [21]. Consistent with these findings, our study demonstrated that short-term wear of HAL lenses does not affect static visual field sensitivity in children with myopia, suggesting that the visual system can adapt effectively to the peripheral defocus of HAL lenses without measurable loss of sensitivity.

Previous studies have concentrated mostly on short-term immediate effects, while the long-term effect on children’s peripheral visual sensitivity needs to be verified. Therefore, this five-year study of wearing HAL lenses provides valuable preliminary evidence to fill this gap. This study supports the safety of long-term use of HAL lenses, which is consistent with the findings of previous short-term studies [15, 21]. Although the HAL group showed numerically slightly higher peripheral sensitivity values, these differences were small and not statistically significant, which is consistent with the findings of Gao et al. [15] This lack of significance could be due to the effect of the lenslets, which is typically below perceptual thresholds in the peripheral regions [22]. However, Ding et al. reported superior central contrast sensitivity in a SVL group compared with a HAL group, as evidenced by a larger area under the log CSF [23]. Long-term wearing of HAL may lead to compensatory responses in the noisy neural environment (peripheral defocus). Persistent peripheral defocus is thought to impair the ability of the primary visual cortex to filter external noise, leading to a poor signal-to-noise ratio in peripheral visual pathways. To continue detecting faint stimuli against this noisy background, the visual system may increase its neural gain, resulting in a more responsive peripheral visual system that behaviorally manifests as a trend toward increased sensitivity. Also, as reported by Huang et al., children who wore SVL for two years showed greater increases in peripheral eye length and more negative changes in peripheral refraction than those who wore HAL lenses [24]. The SVL group exhibited more hyperopic relative peripheral refraction (RPR), but less hyperopic in the HAL group. Lower peripheral hyperopic defocus may contribute to the slightly better peripheral visual field sensitivity observed in children with long-term HAL lenses wear. If confirmed in larger samples, potential adaptive mechanisms could be explored. Therefore, this insignificant improvement is not a true functional enhancement but rather an adaptive adjustment to chronic noise or a consequence of changes in RPR, explaining its subtle, and often subperceptual nature.

In the present study, visual function was assessed using static perimetry with brief stationary light stimuli. However, daily visual experiences are dynamic and involve continuous motion processing, temporal modulation, and complex spatiotemporal integration. Myopia control lenses such as HAL designs introduce peripheral defocus patterns that may interact differently with dynamic visual processing compared to static luminance detection tasks. Therefore, although static perimetric sensitivity was not affected in the current study, future investigations that incorporate dynamic visual stimuli into the measurement paradigm, such as kinetic perimetry, useful field of view, coherence motion detection tasks, or paradigms that include temporal modulation or external noise, may provide complementary insights into the functional impact of myopia management lenses under conditions that more closely approximate real-world vision.

One limitation of this study is that participants in the SVL group were not randomly assigned or recruited, which may introduce potential selection bias. It is possible that children who did not receive myopia control interventions differed in myopia progression. However, the baseline characteristics of the participants across the two groups, including age, sex, SER, and AL, were similar, and there is currently no evidence suggesting that historical myopia progression speed influences automated perimetric sensitivity. Therefore, although residual confounding cannot be completely excluded, the likelihood that selection factors substantially influenced the visual field outcomes appears limited. The second limitation was the relatively high exclusion rate, which can be attributed to extended examination durations, resulting in increased blinking and gaze shifts among children. Increasing the attractiveness of the fixation target by converting it into a dynamic cartoon image may increase fixation interest [25]. However, the reliability parameters and test duration of visual field measurements did not differ between the HAL group and the SVL group regardless of lens type.

## Conclusion

The impact of long-term HAL lenses wearing on static visual field sensitivity in adolescents aged 13–18 years, who started wearing HAL lenses at 8–13 years old, was examined. The study provides a reference for the safety of myopia management spectacle lenses. However, neither younger children nor the effects of dynamic visual stimuli, which better reflect daily visual experiences, were considered in this study. Future research should employ more sensitive detection methods to assess the effects of myopia management lenses on younger children.

## Supplementary Information

Below is the link to the electronic supplementary material.

Supplementary Material 1: Additional file 1: Table S1 GHT scores of the HAL and SVL groups when wearing HAL lenses and SVL, presented as number of subjects (percentage); Table S2 VFI, MD and PSD of the HAL and SVL groups when wearing HAL lenses and SVL. Description of data: Visual field test reliability parameters between groups under both lens conditions, indicating that group and lens type had no significant influence on test reliability. Further details on the visual field–related parameters are available in electronic supplementary material Table S1 and Table S2.

## Data Availability

The datasets used and analyzed for the present study are available from the corresponding authors upon reasonable request.

## Abbreviations

HAL: Highly aspherical lenslets
SVL: Single vision lenses
SER: Spherical equivalent refractive error
GEEs: Generalized estimating equations
OK: Orthokeratology
BCVA: Best-corrected visual acuity
SITA: Swedish interactive threshold algorithm
SAP: Standard automated perimetry
