# Supplementary material for: Effect of the long-term wearing of spectacle lenses with highly aspherical lenslets on visual field sensitivity in children with myopia
Source: BMC Ophthalmol. 2026 Mar 31;26:250. doi: 10.1186/s12886-026-04781-1 (PMC13162386; doi:10.1186/s12886-026-04781-1)
Supplement: Supplementary file 1 — Supplementary Material 1: Additional file 1: Table S1 GHT scores of the HAL and SVL groups when wearing HAL lenses and SVL, presented as number of subjects (percentage); Table S2 VFI, MD and PSD of the HAL and SVL groups when wearing HAL lenses and SVL. Description of data: Visual field test reliability parameters between groups under both lens conditions, indicating that group and lens type had no significant influence on test reliability. Further details on the visual field–related parameters are available in electronic supplementary material Table S1 and Table S2. [file 12886_2026_4781_MOESM1_ESM.docx]

**Table S1 GHT scores of the HAL and SVL groups when wearing HAL lenses and SVL, presented as number of subjects (percentage)**

| GHT score | Group | HAL test lens | SVL test lens | Groups | | Test lens | | Group*Test lens | |
| --- | --- | --- | --- | --- | --- | --- | --- | --- | --- |
| Within Normal Limits | HAL group | 11 (40.74%) | 8 (32%) | 0.552 | 0.169 | | 0.947 | |  |
|  | SVL group | 12 (36.36%) | 9 (28.13%) |  |  |  |  |  |  |
| Borderline | HAL group | 13 (48.15%) | 13 (52%) |  |  |  |  |  |  |
|  | SVL group | 16 (48.49%) | 16 (50%) |  |  |  |  |  |  |
| Outside Normal Limits | HAL group | 3 (11.11%) | 4 (16%) |  |  |  |  |  |  |
|  | SVL group | 5 (15.15%) | 7 (21.88%) |  |  |  |  |  |  |

**GHT: Glaucoma Hemifield Test; HAL: spectacle lenses with highly aspherical lenslets; SVL: single vision lenses.**

**Table S2 VFI, MD and PSD of the HAL and SVL groups when wearing HAL lenses and SVL**

|  | Group | HAL test lens | SVL test lens | Groups | | Test lens | | Group*Test lens | |
| --- | --- | --- | --- | --- | --- | --- | --- | --- | --- |
| VFI | HAL group | 96.07 (0.79) | 95.24 (0.71) | 0.910 | 0.477 | | 0.552 | |  |
|  | SVL group | 95.61 (0.76) | 95.53 (0.58) |  |  |  |  |  |  |
| MD (dB) | HAL group | -2.65 (0.30) | -2.47 (0.32) | 0.178 | 0.267 | | 0.785 | |  |
|  | SVL group | -3.40 (0.49) | -3.10 (0.42) |  |  |  |  |  |  |
| PSD | HAL group | 2.57 (0.25) | 2.41 (0.29) | 0.259 | 0.813 | | 0.284 | |  |
|  | SVL group | 2.80 (0.33) | 3.05 (0.33) |  |  |  |  |  |  |

**VFI: Visiual Field Index; MD: Mean Deviation; PSD: Pattern Standard Deviation; HAL: spectacle lenses with highly aspherical lenslets; SVL: single vision lenses.**
